# Cultural adaptation of the brain health assessment for early detection of cognitive impairment in Southeast Nigeria

**DOI:** 10.3389/frdem.2024.1423957

**Published:** 2024-06-18

**Authors:** Chukwuanugo Ogbuagu, Ekenechukwu Ogbuagu, Obiageli Emelumadu, Uzoma Okereke, Irene Okeke, Godswill Chigbo, Katherine L. Possin, Isabel E. Allen, Elena Tsoy, Richard Uwakwe

**Affiliations:** ^1^Comprehensive Health Center, Nnamdi Azikiwe University Teaching Hospital Neni, Anambra, Neni, Nigeria; ^2^Global Brain Health Institute, University of California, San Francisco, San Francisco, CA, United States; ^3^Department of Family Medicine, Nnamdi Azikiwe University Teaching Hospital Nnewi, Anambra, Nnewi, Nigeria; ^4^Comprehensive Health Center, Nnamdi Azikiwe University Teaching Hospital Nnewi, Anambra, Nnewi, Nigeria; ^5^School of Public Health, University of Port-Harcourt River State, Port-Harcourt, Nigeria; ^6^Department of Biostatistics and Epidemiology, University of California, San Francisco, San Francisco, CA, United States; ^7^Mental Health Unit, Department of Medicine, Nnamdi Azikiwe University Teaching Hospital Nnewi, Anambra, Nnewi, Nigeria

**Keywords:** cultural, adaptation, cognitive test, community, Nigeria, dementia

## Abstract

**Objective:**

The aging population in developing countries demands parallel improvements in brain health assessment services to mitigate stigma, promote healthy aging, and diagnose cognitive impairments including dementia in primary health care (PHC) facilities. The lack of culturally appropriate cognitive assessment tools in PHC facilities delays early detection. This study aims to culturally adapt a brief digital cognitive assessment tool for PHC professionals in Southeast Nigeria.

**Method:**

A total of 30 participants (15 healthcare workers HCW and 15 community members) were selected to be culturally representative of the community. We completed focus groups and pilot testing to evaluate and refine the Brain Health Assessment (BHA) a subset of tools from the Tablet-based Cognitive Assessment Tool (TabCAT) known to be sensitive to cognitive impairment in other settings. We examined BHA subtests across local languages (Pidgin and Igbo) spoken at two geriatric clinics in Anambra State Southeast Nigeria.

**Results:**

Following structured approaches in focus groups, adaptations were made to the Favorites (memory) and Line Length (visuospatial) subtests based on their input. Participants found the new adaptations to have good construct validity for the region.

**Conclusions:**

The BHA subtests showed content validity for future work needed to validate the tool for detecting early cognitive changes associated with dementia and Alzheimer's disease in PHC settings. The use of culturally adapted and concise digital cognitive assessment tools relevant to healthcare professionals in Southeast Nigeria's PHCs is advocated.

## Introduction

The lack of published research makes it hard to quantify the burden of dementia in Nigeria. The reported prevalence of dementia in Sub-Saharan Africa varies widely between 2% and 22% (Olayinka and Mbuyi, 2014) with incidence rates estimated at 13.3 per 1,000 person-years, increasing mortality in parts of transforming Africa (Akinyemi et al., 2022). The pooled crude prevalence of dementia in Nigeria is reported at 4.9%, and higher in women compared to men (Adeloye et al., 2019). Here, the estimated number of dementia cases increased by over 400% over 20 years, from 63,512 in 1995 to 318,011 in 2015 among persons aged ≥60 years (Adeloye et al., 2019).

In 2050, it is projected that the number of older Nigerians will triple to more than 33 million to become the world's 11th-largest older population (United States Census Bureau, 2022). The US Census Bureau projected that in 30 years, 36 African countries will have more than a million older adults and seven countries will have at least 10 million older adults (United States Census Bureau, 2022). Given that older age is the predominant risk for dementia, these predictions buttress the urgency of creating culturally appropriate screening tools that will identify early cognitive changes.

The lack of culturally appropriate cognitive assessment tools remains a challenge to the diagnosis of cognitive disorders. This is especially true in resource-constrained economies like Nigeria with no aging brain health policies. Screening tools translation undertaken without due attention to cultural nuances, as often used in research and practice, is inadequate and produces misleading and inaccurate conclusions (Allden et al., 2009). On the other hand, rigorous cultural adaptation and clinical validation procedures involving partnerships with members of the local community can improve cultural appropriateness (Kaiser et al., 2015; Atilola et al., 2016; Kohrt et al., 2016). Furthermore, assessment instruments that are culturally adapted function better in validation studies than tools that have not been culturally adapted before deployment (Weobong et al., 2009; Ali et al., 2016). A recent survey conducted among healthcare workers (HCW) showed a lack of knowledge of cognitive assessment tools for dementia and Alzheimer's in highly stigmatized communities (Ogbuagu et al., 2023).

A shift from paper and pencil to digital cognitive assessment tests is highly advantageous, particularly for Nigeria experiencing rapid demographic aging and widespread cognitive screening (Thompson et al., 2023). Digital tests improve efficiency, accuracy, and accessibility, making the assessment process smoother and reducing logistical challenges and the subjective nature of current standardized cognitive assessment methods (Staffaroni et al., 2020). They enable remote testing, increasing access for individuals in rural or underserved areas, and provide instant feedback for timely intervention (Chan et al., 2021). Overall, digital cognitive assessments can significantly enhance diagnostic accuracy and support equitable brain health assessment services in PHCs in Nigeria.

Adaptation and/or validation approaches have been used successfully to develop screening tools for psychoemotional disturbance and depression for use among adults in primary care settings in Nigeria (Abiodun, 1993; Omigbodun et al., 1996; Adewuya et al., 2018), but digital cognitive assessments are lacking. The BHA demonstrates 100% sensitivity and 84% specificity in identifying dementia and mild cognitive impairment (MCI), including MCI resulting from various causes (Possin et al., 2018). Its subtests offer effective and reliable assessments of neurocognition, which are crucial for making accurate differential diagnoses. The BHA tests have been both culturally adapted and clinically validated in other populations (Rodríguez-Salgado et al., 2021; Gianina et al., 2023). It is a psychometrically robust and clinic-user-friendly cognitive screening tool enabling easy and secure access to data for both research and clinical applications (University of California San Francisco, 2024). It also has an expanding reach as a cognitive testing application for research and clinical use, with robust and dynamic scoring and reporting, informative resources, Health Insurance Portability and Accountability Act (HIPAA) compliance, and portable (University of California San Francisco, 2024). This framework for protecting patient health information and ensuring data privacy and security applies to Nigeria and the US. This screening tool includes memory, executive and speed, language, visuospatial, and motor speed subtests and was developed for the evaluation of mild neurocognitive disorders in primary care and other everyday clinical settings. This study has the potential to stimulate cognitive assessment in other countries.

The objective of this study was to adapt and perform preliminary cultural validation of the BHA tests for use by healthcare professionals working in two PHCs for older adults in Southeast Nigeria.

## Method

### Study design

The study design followed the guidelines proposed by the International Test Commission (ITC) (International Test Commission, 2017). Focus groups and pilot testing were held between November and December 2022. The study received ethical approval from Nnamdi Azikiwe University Teaching Hospital (NAUTH) in Nnewi, Nigeria (Nnamdi Azikiwe University Teaching Hospital Anambra State Nigeria, 2024). All the participants were adequately briefed about the study before obtaining informed consent from them.

### Participants

Fifteen adult participants from the community and fifteen healthcare workers were enrolled and split into three smaller subsets. Using purposive sampling, we selected through personal contacts and advertising in the community participants with a basic level of knowledge and experience with electronic devices, equal gender balance, and the majority with trilingual fluency in English, Pidgin, and Igbo. The healthcare workers served as expert reviewers, according to ITC guidelines, offering native indigenous knowledge of the cultures and languages involved as well as general principles of testing. To ensure that the participants were reasonably cognitively intact, any participant with a known history of diabetes, hypertension, stroke, any known neurologic problems, or HIV as well as known those excessively using alcohol, smoking, or those with head injuries were excluded. The validation participants involved four different cadres of HCWs and four socioeconomic classes from the community (Table 1).

**Table 1:** Distribution of cultural validation participants.

**Variable**	**FGD**		***p*-value**
	**Healthcare professionals (*n* = 15)**	**Community representative (*n* = 15)**	
Age, M (SD)	64.0 (15.9)	64.1 (11.9)	0.984
Years of education, M (SD)	9.5 (0.4)	10.1 (0.6)	0.559
**Gender**, ***N*** **(%)**			
Males	7 (46.7)	8 (53.3)	0.435
Females	8 (53.3)	7 (46.7)	
**Participants subsets**, ***N*** **(%)**			
Physician	5(33.3) Artisan	5 (33.3)	
Nurses	5(33.3) Motorcyclist	5 (33.3)	
Pharmacists	2 (13.3) Rel. leader	2 (13.3)	0.327
CHEW	3 (20) Farmer	3 (20)	
**Total**	15 (100)	15(100)	

Rel. leader, religious leader; CHEW, community health extension workers.

### Setting

Focus groups were assembled at Nnamdi Azikiwe University Teaching Hospital (NAUTH), a tertiary healthcare facility located in Nnewi, Anambra State, Nigeria that offers comprehensive medical services in all subspecialties including PHC. The work was conducted at two geriatrics clinics within this facility.

### BHA tests

The following tests were included in this adaptation. Favorites is a test of associative memory. Match is a test of executive functions and processing speed. Line Orientation and Line Length examine visuospatial ability through the examination of the orientation of lines and line length, respectively. Animal Fluency is a test of semantic word generation. Quick Tap tests tapping speed. Detailed descriptions of all tests are below along with the results, and additional details are available online (University of California San Francisco, 2024). We excluded the questionnaire on brain health survey which is a test to be completed by an informant who knows the examinee very well.

Favorites is a test of associative memory for visual and verbal information in which examinees are asked to remember faces paired with a food and animal word. The task consists of two learning trials, each followed by an immediate recall trial, and a 10-min delayed recall and delayed recognition trial.

Animal Fluency is a widely used test of language generation. Examinees are asked to generate as many animals as possible in 1 min, and the total correct is recorded. The examinee is presented with a consistent legend displaying numbers 1 through 7, each accompanied by distinct, abstract images. These images are positioned both just below the respective numbers and in a different sequence along the bottom of the screen. The instructions guide the examinee to promptly tap the matching picture at the bottom of the screen whenever a number is displayed in the center of the screen. Following each response, a new number is presented.

Motor Quick Tap provides a measure of motor speed and dexterity. During task completion, the tablet should be placed flat on a table or surface. The examinee should first rest their non-dominant hand on the tablet surface with their index finger placed within a designated area on the screen. When prompted, they should tap their non-dominant index finger repeatedly, as fast as possible, until the end of the trial. There are 5 trials of 10 s each. The trials begin with a tone that signals the start, and another tone that signals the end. The designated tapping area also changes color from green to red to signal the trial start and stop.

Line Length is a test of visuospatial skills and was developed as a potentially more accessible alternative to Line Orientation for examinees with language difficulties. Examinees are shown 2 parallel white lines of differing lengths on a navy background. Examinees are asked to tap the longer line of the two.

Line Orientation is a test of visuospatial skills and is modeled after the Benton Judgement of Line Orientation task. Examinees are shown 3 lines on a dark navy background; 1 white line between and shifted vertically above 2 shorter orange lines. One orange line is shown parallel to the white line, whereas the other orange line is presented at a different angle. Examinees are asked to tap the orange line that is parallel to the target white line.

### Exclusion criteria

Participants were excluded for known history of diabetes, hypertension, stroke, psychiatric problems, or HIV infection. We also excluded any reported history of active or recent substance abuse, or neurologic or systemic diseases that are meaningfully contributing to cognitive, behavioral, or functional decline. Also, any lifetime history of psychiatric or developmental disorders (e.g., schizophrenia, autism spectrum disorders). Any sensory or motor impairments that may compromise testing procedures and residence in a skilled nursing facility were also excluded.

### Protocol/procedure

#### Step 1. Community entry

The study team met with 40 potential participants in a town hall-style meeting where everyone gathered and introduced them to the collaborative work plan, integrating them into the project, in compliance with Consolidated Criteria for Reporting Qualitative Research (COREQ) techniques. After this presentation, eligibility was checked, and 30 participants qualified and enrolled in the project.

#### Step 2. Pilot testing round one

The 15 healthcare workers and 15 community participants met separately in their two respective groups, each with 3 iPads and a psychometrist facilitator, and took turns taking the TabCAT tests. They discussed comfort, familiarity, and feasibility of using the device. They did not discuss the details of the tests at this stage.

#### Step 3. Cultural review, proposed adaptations, and translation focus groups

Three focus groups of 10 people, mixed with an equal number of healthcare workers and community participants in each group and each with a psychometrist facilitator, reviewed and discussed each test in detail for cultural appropriateness and needed adaptations for comfort and familiarity across the 3 group members. They also proposed translations to the tests.

#### Step 4. Group consensus meeting on cultural adaptations and translations

All 30 participants with the 3 facilitators discussed the results together to come to a consensus. For both Step 3 and Step 4, the groups were guided by psychometricians and followed a structured approach to evaluate and consider adaptations of the tool's relevance, appropriateness, and effectiveness across diverse cultural Igbo contexts. They engaged in discussions and activities to evaluate the tool's cultural sensitivity and applicability with consideration of potential biases, language nuances, and cultural factors that may influence the interpretation or performance of the test in the community. The sessions were recorded. Insight gathered from these focus groups informed adjustments to enhance the tool's appropriateness and potential validity across diverse populations in Nigeria. A session was dedicated to back translation which was the “re-translation” of the translated version back into the original language and subsequent comparison of the original version and then back translation - to ensure that the construct and content validity is maintained. Some diverse cultural Igbo contexts questions were raised for example, the importance of phonetics and accent marks in differentiating words with multiple meanings like àkwá- egg; ákwà-cloth; àkwà-bed; ákwá- cry, and words with no translations in native languages like parallel - pararelu.

#### Step 5. Pilot testing round two

Each participant individually took the tests again, observed by a study doctor or psychometrist. After completing the test, they were asked “Was there any difficulty in understanding and taking the test?” “What are the challenges in handling the electronic device while taking the test?” “Were there any instructions or stimuli (eg, words, faces, animals) that sounded strange or unfamiliar?”. Some of the comments were “The test was very friendly and easy to understand”, and “The device provides clear directives on what to do next”.

#### Step 6. Final expert review

The translated and back-translated versions were reviewed for cross-cultural equivalence by members of the expert panel, specifically, we selected four doctors from the healthcare worker group with expert knowledge in the field of cognitive assessment, two of whom were professors (one of mental health and one of public health) and the other two were senior clinicians. They responded that “The test tool showed content familiarity following adaptations and appeared useable in PHCs.”

### Data analysis

The focus group sessions were recorded and transcribed. The panel of experts reviewed the notes to pull out the key themes and recommendations. Thematic analysis was employed to identify recurring patterns, themes, and cultural nuances emerging from participants' responses arising from the different dialects (Braun and Clarke, 2006). We explored cultural factors influencing the tool's relevance, acceptability, and effectiveness for content validity across various cultural contexts.

## Results

### TabCAT tasks cultural validation themes by cognitive domain

1. Memory: favorites (verbal and visual associative memory):

Response:

***Physician**: The animals and food should be the types that are relevant to the environment and the age group to be tested and should be ideally the common ones found in our environment. Examples of common animal types are Ewu* = *Goat, and Atulu* = *Sheep. Food types should include for example Oloma* = *Orange Agwa* = *Beans. Most importantly, to use animals familiar with our environment which was the problem with MoCA that used unfamiliar animals in their tests. It is also important to distinguish the spelling with phonetics and signs for a word that have the same spellings but different meanings and almost the same pronunciation eg Egbé* = *Hawk; Égbè* = *Gun*.

*The faces are there for the examinee to remember and pose no threat to the content of the tool*.

***Nurses**: It would be ideal to replace the food items with local delicacies common in our environments to remove possible difficulties in remembering foreign food items. For example, we could replace lettuce with Ube* = *Pear*.

***Pharmacists:*** *The animals in the test were not common in our environment even though seen on television but it would be fair to use animals commonly seen around like Ewu*= *Goat!*

***Community Health Extension Workers (CHEW)**: The translation of the names of the animals would be better in the Igbo native dialect to encourage recall by the patients*.

***Artisan**: We need familiar animals and possibly faces of past Nigerian leaders, not the current ones*.

***Religious and Community Leader**: We are not familiar with these food types. We would appreciate foods grown in our community like Melon* = *Egusi, and Local apple* = *Udala*.

2. Language: animal fluency (categorical verbal fluency):

***Responses:***

***Physician**: The animal response by the examinee to be accepted should include common and uncommon ones provided it falls into the category of an animal whether mythical or real*.

***Artisan/Community leader/Religious leader/Farmer**: Some animals with no Igbo translations should be accommodated and scored for example Antelope is translated to “Antelōpu in Igbo”*.

***Farmers**: The animal responses should include the ones most commonly seen in our clime but known to exist in other communities where the tool is to be used*.

3. Executive function: match (processing speed and executive functions):

***Response:*** *Physician: This does not require any amendment as the images and numbers were to be memorized by the examinee and come out in ideal timing*.

***Nurses**: This does not require any adjustment. It is perfect with the correct timing*.

***Farmer:*** *The objects assigned with specific numbers need no modification. It is well-understood and easy to follow*.

***Motorcyclist:*** *The objects and numbers are clear for anyone to understand and need no modification*.

4. Motor: Quick tap

***Response:*** *Physician: The prompts and instructions are clear with no ambiguities*

***Nurse:*** *This instruction is clearly stated in the tool. Very clear to understand*.

***Religious leader***: *This one is easy to follow and well-designed*.

***Farmer:*** *This part is clear, and everyone can understand it*.

5. Visuospatial: line length.

***Response:*** *Physician: The lengths are well thought out and the idea of tapping is good to stimulate the examinee. There would be no need to modify any part of the test, but the emphasis should be on the keyword “length” to mean “ogologo” in Igbo*.

***Nurse**: This is a good test with ease of performance by the patient. The color change makes it engaging to retain the patient's attention*.

***Motorcyclist**: The lengths are almost the same, so emphasis should be on the word “length” to mean “ogologo” in Igbo*.

6. Visuospatial: line orientation.

***Physician**: The word parallel is confusing to translate in Igbo. With the different dialects, some translated “parallel” as “dika”, ‘'dabere”. The consensus was to emphasize the translation of parallel as “yiri” for universality in understanding*.

***CHEW**: It would be nice to translate the word “parallel” as “dinē ka” but “yiri” would be the best option, and there would be a need to observe the patient's face to make sure he/she understands the action word*.

***Religious leader**: The word “parallel” needs to be translated verbatim into “pararellu” with possible additional illustration by the examiner to ensure that the patient understands the action word*.

The data gathered showed a mean age of 64 years for the healthcare workers (HCW) and community representatives while 53.3% were female and the mean years of education attained were 9.5 and 10.1 for the HCW and community representatives respectively (Table 1).

## Discussion

A total of thirty bilingual or trilingual (English, Igbo, and Creole English known as Pidgin) healthy volunteers were enrolled to participate in the cultural validation, with both genders equally represented. Against this backdrop of trilingualism and diversity that represented the target population for the tests, we were able to address the nuances in phonetics, dialect, and understanding while preserving the content of the tests.

Most of the tests were found to be culturally appropriate, and major changes were only required for two of the six subtests: Favorites and Line Orientation. For Favorites, the panel of experts suggested replacing some of the existing animal and food types with more familiar ones from the community. For Line Orientation, the concept of parallel was challenging to translate and recommendations were made for terminology. It is also possible that this test will not work for our target population and the alternative Line Length will be more appropriate, in future work. This will be examined in future validation work. It was only words that had cultural (and linguistic) limitations. The other sub-tests were culturally entirely suitable maybe because of the literacy and numeracy level in the study community. This study supports culturally adapting screening tools which remains the vital first step toward improving case detection (Kaiser et al., 2019), before the deployment for use in the community.

In summary, we found evidence that the translated TabCAT tests show content validity in Southeast Nigeria. Local experts felt that the tool reflected the general characteristics of the original version. Participants felt that the tests would be appropriate and useable in PHCs and are suitable for validation as a screening test for dementia, once normative data are obtained, a procedure that will also allow us to examine performance characteristics in a healthy population.

It is imperative to prioritize the implementation of culturally validated cognitive assessment tools in PHC facilities, especially in regions experiencing rapid demographic aging such as in Southeast Nigeria. This proactive approach not only facilitates early detection and intervention for cognitive impairment but also fosters community awareness and acceptance, combatting the stigma often associated with aging-related conditions like dementia. Furthermore, investing in healthcare infrastructure tailored to local needs strengthens healthcare systems overall, promoting holistic wellbeing among elderly populations (Alzheimer's Disease International, 2019). By equipping healthcare professionals with user-friendly tools tailored to the local context, we can significantly enhance brain health assessment services and promote overall wellbeing among the elderly population (National Institute on Aging, 2021). This recommendation aligns with global efforts to improve healthcare accessibility and quality, ensuring that vulnerable communities receive the support they need for healthy aging. The study is sensitive to the nuances around context and construct associated with cognitive tests and is a culture-fair assessment like the RUDAS multicultural cognitive assessment scale (Storey et al., 2004).

## Limitations

There may have been unforeseen cultural factors or biases capable of influencing results such as vocabulary and nuances associated with sample selection as a representative of a single culturally similar group. Familiarity with technology could constitute a gap in the application with selected target populations.

## Conclusion

The adapted BHA tests demonstrated sufficient content validity and showed advantage, particularly for regions experiencing rapid demographic aging that may require widespread cognitive screening. Further examination and validation for detecting cognitive changes associated with dementia may be required depending on the locality.

## Data availability statement

The datasets presented in this study can be found in online repositories. The names of the repository/repositories and accession number(s) can be found in the article/Supplementary material.

## Ethics statement

The studies involving humans were approved by Nnamdi Azikiwe University Teaching Hospital Ethics Board. The studies were conducted in accordance with the local legislation and institutional requirements. The participants provided their written informed consent to participate in this study.

## Author contributions

CO: Writing – original draft, Writing – review & editing, Conceptualization, Project administration. EO: Methodology, Writing – original draft. OE: Project administration, Supervision, Writing – review & editing. UO: Project administration, Writing – original draft. IO: Project administration, Writing – original draft. GC: Data curation, Methodology, Validation, Writing – review & editing. KP: Conceptualization, Methodology, Writing – review & editing. IA: Data curation, Validation, Writing – review & editing. ET: Conceptualization, Writing – review & editing. RU: Supervision, Writing – review & editing.
