# Supplementary material for: Cultural adaptation of the brain health assessment for early detection of cognitive impairment in Southeast Nigeria
Source: Front Dement. 2024 Jun 18;3:1423957. doi: 10.3389/frdem.2024.1423957 (PMC11285541; doi:10.3389/frdem.2024.1423957)
Supplement: Supplementary file 1 [file Data_Sheet_1.pdf]

Supplementary Material - Cultural Adaptation of the Brain Health Assessment for Early Detection of Cognitive Impairment in Southeast Nigeria - Igbo Language - Nigeria (September 2023)

Chukwuanugo Ogbuagu, MD, PhD; Richard Uwakwe, MD, FWACP; Uzoma Okereke MD, MPH

The commands and instructions were independently translated by a team of Primary Healthcare Physicians and a Psychiatrist from English to Igbo, accommodating the different Igbo native-speaker dialects and cultural nuances with pronunciation and meaning. We adopted the central Igbo (Igbo Izugbe) language dialect in the translation while accommodating other dialects. Emphasis was made on adding phonetics (uda olu) to ensure correct pronunciation of the word to maintain the meaning and construct. We all arrived at a consensus that a locally trained examiner would be necessary when administering the test. From the English version, we selected high-frequency words that we judged would be familiar to all people in the population who may take the test irrespective of the different dialects. We removed the most prototypical words from each category (e.g., snake which in Igbo could be called Agwo, Eke, Abuani), and any words that are commonly used to refer to both animal and another thing (e.g., Hawk=Égbé, Gun=Ēgbè), and any words that are very long, compound, or phonetically complex. The forms are also balanced for difficulty by word frequency and syllable count.

The list of words (fruits, vegetables, and animals) was generated by the panel of experts during the cultural validation of a cognitive assessment tool independently, and consensus was arrived at before adoption because a word in the Igbo language could have more than one meaning. As such phonetics and marks are usually used to differentiate the meaning. For example, àkwá-egg; ákwà-cloth; àkwà-bed; ákwá- cry. To ensure appropriateness in understanding the word, the pronunciation of the word is very important to ensure correct phonetical interpretation of the word

for cultural appropriateness. The final forms were created based on the consensus between the panel of experts and the Psychometricians, regarding the above criteria.

Animals

Form A		Form B		Form C		Form D	
English	**Igbo**	English	**Igbo**	English	**Igbo**	English	**Igbo**
Eagle	Ugo	Tiger	Agu	Monkey	Enwe	Sheep	Atụlụ
Fish	Azụ	Monkey	Enwe	Pig	Ezi	Cow	Efi
Dog	Nkita	Lizard	Ngwere	Duck	ọbọgwụ	Rat	Oke
Vulture	Udene	Frog	Awo	Goat	Ewu	Turtle	Mbe

Fruits and Vegetables

Form A		Form B		Form C		Form D	
English	**Igbo**	English	**Igbo**	English	**Igbo**	English	**Igbo**
Coconut	Akụ oyibo	Banana	Unere	Mushroom	Elo	Yam	Ji
Orange	Oloma	African star apple	Udala	Maize	Oka	Bitter leaf	Onugbu
Garden egg	Ańara/afufa	watermelon	Anyu mmiri	Casava	Jigbo	pepper	Ose
Plantain	Ojoko	Kolanut	Oji	Bread fruit	Ukwa	Rice	Osikapa

Literature we used when choosing words:
https://www.igboguide.org/HT-vocabulary.htm
https://ezinaulo.com/igbo-lessons/vocabulary/animals/
